# Revising economic measures of economic activity to reflect human and environmental health

**DOI:** 10.2471/BLT.23.290662

**Published:** 2024-02-22

**Authors:** Mark Hanson, Neena Modi, Paul Allin

**Affiliations:** a Academic Unit of Human Development and Health, University Hospital Southampton, Tremona Road, Southampton SO16 6YD, England.; b Faculty of Medicine, School of Public Health, Imperial College London, London, England.; c Department of Mathematics, Imperial College London, London, England.

The work of the World Health Organization (WHO) Council on the Economics of Health for All^1^ is welcome in that it seeks to transform economies to deliver what is truly important to humanity and our planet. We agree with the council’s view that policy-makers’ reliance on gross domestic product (GDP) as a measure of economic growth and prosperity is flawed because it includes outputs from activities that harm human and environmental health but excludes those fundamental to health that do not generate financial outputs. However, we question the recommendation to move away from reliance on GDP towards additional measures such as the Genuine Progress Indicator or dashboards of multiple metrics. The components that define GDP are currently under review through the System of National Accounts, the internationally agreed set of recommendations on how to compile measures of economic activity. The review offers an opportunity for much-needed change. We propose that the definition of GDP should be revised to incorporate measures of human and environmental health, and that this calculation should be through a net rather than a gross summary measure of productivity, that is, one which includes the costs to health instead of focusing solely on the economic impacts of activities.

To explain our perspective, we consider the nature of the System of National Accounts. The reality that planetary and human health and the economy are interrelated is widely understood by policy-makers but fails to be reflected in the System of National Accounts. At the centre of the accounts are measures of the production of goods and services,^2^ a limited economic view that focuses on transactions measurable in financial terms. This view proscribes a production boundary of what is included – which, for example, includes paid childcare services but excludes the parental care of children even though such care is a crucial contributor to future human capital. Similarly, GDP growth drives corporate and government behaviour around the world. Yet the international community and policy-makers alike have long recognized that the production and consumption of industrial outputs such as tobacco-based products and unhealthy foods, while increasing GDP, also damage health directly and indirectly such as through environmental pollution or exacerbation of climate change. Persistent adherence to GDP growth as a goal is contributing to worsening population and planetary health worldwide.

The *System of national accounts 2008* acknowledges that such excluded and hence invisible activities are productive in an economic sense, but justifies their omission because the inclusion of large non-monetary flows, together with monetary flows, can obscure what is happening in markets and reduce the analytic usefulness of the data.^2^ This statement reflects outdated concepts of economic worth and cannot be a justification for continuing with the current situation. Additionally, other widely held misconceptions contribute to the persistent exclusion of measures of health from GDP. The first misconception is to cling to the association between economic growth and population health as justification for the former taking precedence over the latter, and failing to recognize that this association does not reflect a causal relationship. This point is shown by the current reversal in high-income countries of the continuous improvement in population health that characterized the twentieth century. The second major barrier to conceptual progress is the continued focus on access to health services through emphasis on the need for universal health coverage, in the mistaken view that health care is the primary driver of health. Yet health care explains only around half of the variance in health in low-income countries and no more than about 20% in high-income countries,^3^ because other factors such as education, nutrition, housing, equity and agency and working conditions play major roles in affecting health. Recognizing the centrality of population health to economies parallels the need to incorporate value to activities that promote environmental and planetary health rather than delegating them to subsidiary or satellite accounts^4^ that may record the level of such activities as greenhouse gas emission but that, because they are subsidiary, may have lower priority than GDP in influencing policy.

The current definition of GDP could potentially change because the *System of national accounts 2008* has undergone five revisions since 1953, with a sixth ongoing under the auspices of the United Nations (UN) Statistics Commission.^5^ However, national accounts methodologists are leading the revision and are largely detached from perspectives beyond macroeconomics and monetary policy. No fundamental change to the hegemonic position of GDP as currently defined, and serving as the measure of societal progress, growth and prosperity, appears to be in prospect.

Alternative measures of progress have been published, such as the Human Development Index,^6^ which includes Gross National Income (a variant of GDP) or dashboards with multiple indicators to be read alongside GDP.^7^ The recent announcement by the Secretary-General of the UN added further impetus to the drive to go beyond GDP in assessing global development. However, rather than modifying GDP, the statement proposes to outline a path to develop complementary metrics that more fully recognize what matters to people, the planet and our future.^8^ This statement, though laudable, fails to reflect the realities of political decision-making that are driven by a concentrated focus on GDP. The WHO Council on the Economics of Health for All supports the concept of such a dashboard, focused on a range of metrics that track progress across core societal values, above and beyond the narrow, static measure of GDP,^1^ suggesting that metrics on sustainable development goals' (SDG) progress might serve the purpose. However, as no countries are on track to meet SDG goals, this avenue is unlikely to have impact.^9^

Given the dominance of GDP in driving government behaviour, using wider measures of economic activities in a dashboard alongside an essentially unchanged GDP to devise economic policy might lead policy-makers to prioritize one measure over another, rather than take the required holistic view. Our proposal is aimed at challenging the narrow view of the economy embedded in the *System of national accounts 2008* and likely to be carried forward in the next planned version. Our vision is that the document should address the primary purposes for which it is designed, that is economic analysis, decision-taking and policy-making.^2^ Incorporating measures of population and environmental health into GDP would force governments to recognize that economic resilience is impossible if they fail to reverse the profound deterioration in human health that is occurring, for example with increasing prevalence of obesity and noncommunicable diseases, falling life expectancy in many populations and increasing environmental despoliation.

There may be global recognition that the future System of National Accounts headline measure should be net rather than gross domestic product, and that the consumption of natural resources should be part of the calculation of costs deducted from GDP, rather than confining this calculation to the depreciation of plant, as is currently the case. However, doing so does not address the problem that GDP does not recognize the value of human and environmental health to the economy. A distinction exists between defining a measure and using it to set targets and develop policy. We cannot expect corporate and government decisions to improve while market-based GDP remains dominant. Leaving national account methodologists to determine the way in which to measure economic and societal performance, and assuming that public and private institutions will prioritize acting in the best interest of the population is dangerous and, as the slow progress achieved in tackling climate change shows, likely to be ineffective. We are also concerned that developments beyond traditional GDP will only receive consideration by the UN Statistics Commission after the current revision is complete in 2025, and only through the development of an integrated statistical system for inclusive and sustainable well-being.^10^ Thus, GDP will continue largely unaltered as a measure of economic performance for the near future during which, on current trajectories, population and environmental health will continue to worsen.

We therefore propose three complementary actions. The first is to challenge the existing assumptions of the *System of national accounts 2008*, with its insistence that population health is not an economic factor and not relevant to economic analysis. We suggest that population health is recognized as an output, a product of activities that contribute to GDP, such as the investment that is (currently unremunerated) parenting. Redefining the System of National Accounts in this way will not be easy nor quick. Existing indicators, such as the Health Index for England^11^ and many national and international metrics, provide practical utility in the short term.

Second, building on the suggestion of netting-off the environmental impacts of economic outputs, we see benefits in presenting positive and negative health contributions separately. The headline measure should be the version of GDP in which activities damaging to population and environmental health have been deducted from the gross product.

Third, as highlighted in the 2009 report of the Commission on the Measurement of Economic Performance and Social Progress, discussion of societal values, for what we, as a society, care about and whether we are really striving for what is important^12^ is long overdue. The world needs a shared understanding of what constitutes progress, as this can no longer be ever-increasing growth. Such a vision is emphasized in the final report of the WHO Council on the Economics of Health for All.^1^ A good place to start would be to ensure that population health is properly included in a revised System of National Accounts.
